# Transcriptome profiling of the flowering transition in saffron (*Crocus sativus* L.)

**DOI:** 10.1038/s41598-020-66675-6

**Published:** 2020-06-15

**Authors:** Jing Hu, Yuping Liu, Xiaohui Tang, Huajing Rao, Chaoxiang Ren, Jiang Chen, Qinghua Wu, Yi Jiang, Fuchang Geng, Jin Pei

**Affiliations:** 1 State Key Laboratory of Traditional Chinese Medicine Resources Research and Development, Chengdu University of Traditional Chinese Medicine, Chengdu, 611137 China; 2 New Zealand Academy of Chinese Medicine Science, Christchurch, 8014 New Zealand; 3 The Good Doctor Pharmaceutical group co. LTD, Mianyang, 622650 China

**Keywords:** Molecular biology, Physiology, Plant sciences

## Abstract

Saffron, derived from the stigma of *Crocus sativus*, is not only a valuable traditional Chinese medicine but also the expensive spice and dye. Its yield and quality are seriously influenced by its flowering transition. However, the molecular regulatory mechanism of the flowering transition in *C. sativus* is still unknown. In this study, we performed morphological, physiological and transcriptomic analyses using apical bud samples from *C. sativus* during the floral transition process. Morphological results indicated that the flowering transition process could be divided into three stages: an undifferentiated period, the early flower bud differentiation period, and the late flower bud differentiation period. Sugar, gibberellin (GA_3_), auxin (IAA) and zeatin (ZT) levels were steadily upregulated, while starch and abscisic acid (ABA) levels were gradually downregulated. Transcriptomic analysis showed that a total of 60 203 unigenes were identified, among which 19 490 were significantly differentially expressed. Of these, 165 unigenes were involved in flowering and were significantly enriched in the sugar metabolism, hormone signal transduction, cell cycle regulatory, photoperiod and autonomous pathways. Based on the above analysis, a hypothetical model for the regulatory networks of the saffron flowering transition was proposed. This study lays a theoretical basis for the genetic regulation of flowering in *C. sativus*.

## Introduction

*Crocus sativus* L., commonly called saffron, is a perennial stemless herb belonging to the family *Iridaceae* (monocots), which is widely distributed in Iran, Spain, Greece, Italy and Nepal^1^. Due to the triploidy of its chromosomes, this plant produces sterile flowers and reproduces asexually by corm nutrition. Saffron was introduced to China from abroad, passing through Tibet, and has been successfully cultivated in many of its provinces, such as Shanghai, Zhejiang, Sichuan and Anhui, since the 1970s. The flower, the most valuable part of saffron, consists of six tepals, three stamens and three stigmas. Among these, the stigma is widely used as a spice or coloring and flavoring agent in both the agro-food and cosmetic industries^2^. The stigma is also used as a medicine due to its important pharmacological efficiency^3^. Thus, saffron is greatly required worldwide due to its wide use. However, in recent years, the saffron flower has experienced increased incidences of withering, rotting, and delayed flowering, which has severely affected the quality and quantity of its stigmas and restricted the sustainable development of the saffron industry. Therefore, this study on the molecular regulatory mechanisms of the saffron flowering transition is particularly urgent and important for understanding and solving the problems related to saffron flowering.

The complex process of the flowering transition is coregulated by both the external environment and the internal factors in plants to ensure flowering at an appropriate time^4^. In the model plant *Arabidopsis thaliana*, the flowering transition was found to mainly involve six regulatory pathways: the vernalization, photoperiod, gibberellin, sugar metabolism, autonomous and age pathways^5,6^. These pathways converge to regulate the expression of flowering-related genes, such as *FLOWER LOCUS T* (*FT*), *CONSTANS* (*CO*), *SUPPRESSOR OF OVEREXPRESSION OF CO 1* (*SOC1*), *LEAFY* (*LFY*), *APETALA1* (*AP1*), *APETALA2* (*AP2*) and *APETALA3* (*AP3*), which irreversibly induce the transition from the vegetative meristem to the floral meristem^6,7^. The above pathways also play important roles in the regulation of flower induction in other plants. Research has shown that sugar, as both the energy substance and the signal molecule, positively mediates the flowering transition of grape^8^. IAA could accelerate the flowering process in *Rosa chinensis*, but ABA inhibits the process by interacting with sugar signals^9^. It has been extensively reported that vernalization regulates flowering in cereals^10–12^ and beets^13^, and a series of vernalization-related genes, including *VRN1*, *VRN2*, *VIN1* and *VIN2*, have been identified in wheat^14^. Rong Zhou *et al*.^15^ identified and confirmed the sesame *CO-like* (*COL*) gene family from sesame genome data. The *CO* gene encodes a B-box zinc-finger transcription factor, which plays a central role in the photoperiod response and flowering regulation in *Arabidopsis*^16^. In addition, studies in other species, such as soybean, cotton, potato, and maize, also showed that the photoperiod pathway largely affects plant flowering^17–20^.

Although a few studies have reported on saffron EST, transcript data and functions of some genes^21–27^, most of which focused on apocarotenoid biosynthesis, corm sprouting and stigma development, little reported about the regulatory mechanisms of the flowering transition in saffron. Thus, in this study, a transcriptomic analysis was performed by high-throughput sequencing. The differentially expressed genes (DEGs) related to the flowering transition during the three stages were analyzed. Additionally, the morphological changes in the saffron flowering transition were observed, and changes in starch, soluble sugar and endogenous hormone contents were measured to comprehensively understand the regulatory networks of the saffron flowering transition. The results of this study lay a foundation for future studies and cultivation efforts, especially for the flowering of *Crocus sativus* L.

## Results

### Morphological characteristics of the saffron flowering transition

Based on the morphological changes in the saffron apical bud meristem from vegetative to reproductive growth, we divided the continuous growth process into three stages: flower bud undifferentiated period (DS), early flower bud differentiation (BS), and late flower bud differentiation (FS).

**Figure 1:**
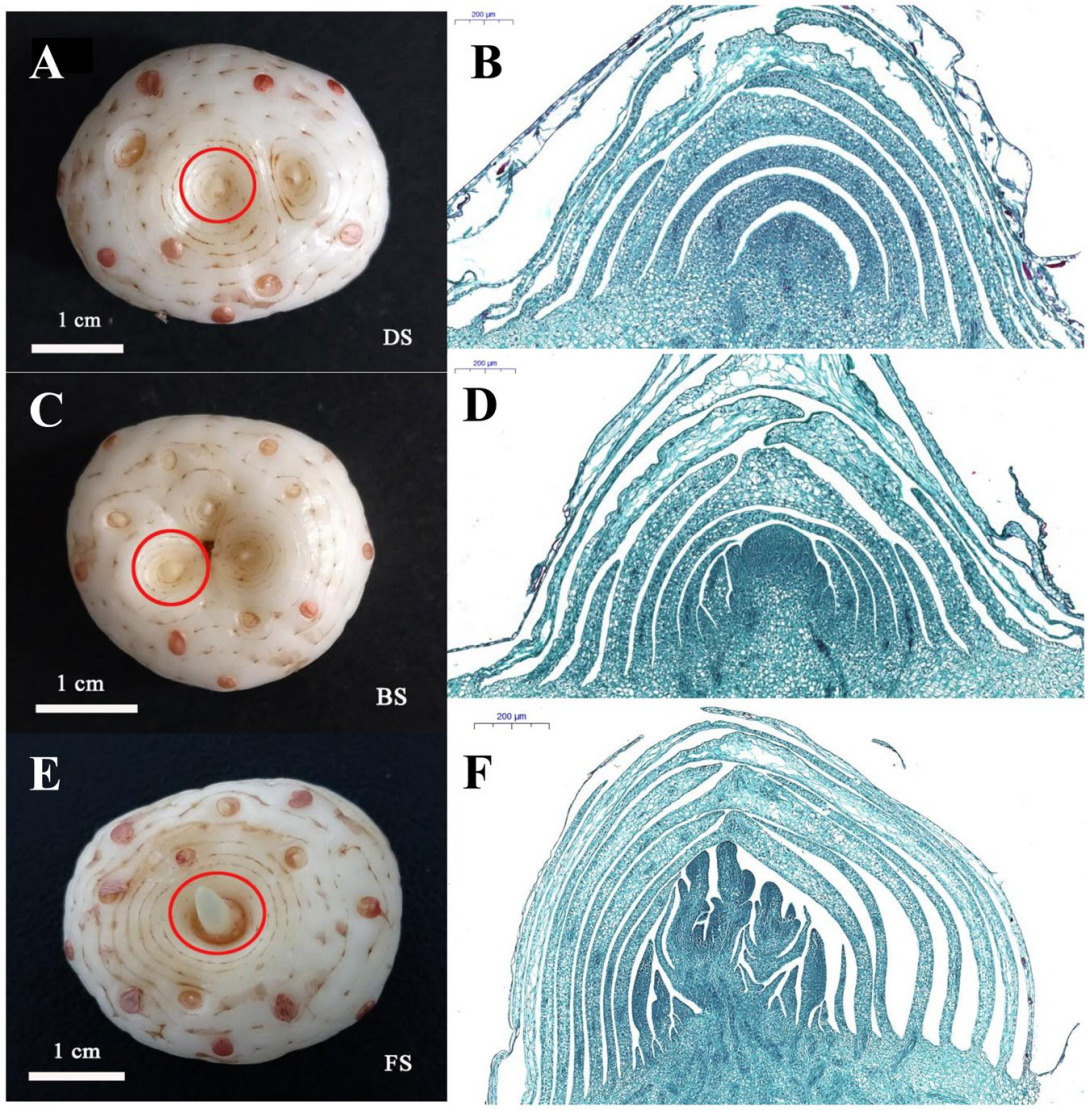
Morphological characteristics of saffron apical bud during floral transition process. (**A,B**) Shown the flower bud undifferentiated period (DS); (**C,D**) Shown the early flower bud differentiation (BS); (**E,F**) Shown the late flower bud differentiation (FS).

In the undifferentiated period, the saffron flower bud was small, less than or equal to 1 mm in length, and the apical growth point appeared semi-conical (Fig. 1A,B). This period was also regarded as the vegetative growth stage because the saffron was gradually breaking dormancy and the floral primordium had not yet formed. At the early flower bud differentiation stage, the length of the flower bud was approximately 1.5–2.0 mm, the growth point had been obviously raised and perianth primordia began to appear, indicating that the saffron had transformed from vegetative to reproductive growth (Fig. 1C,D). In the late flower bud differentiation stage, the flower bud was longer than 3 mm. The differentiation region of the inner bud had become wider and elongated, and the pistil primordia had begun to differentiate (Fig. 1E,F).

## Sugar and hormone contents during the flowering transition process

**Figure 2:**
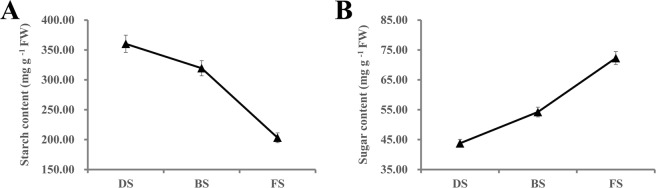
The starch and soluble sugar contents of apical buds during the flowering transition process in saffron. (**A**) starch content and (**B**) soluble sugar content. Values are means of three replicates ± SE.

The levels of starch and soluble sugar in the apical buds were measured at three stages during the flowering transition (Fig. 2). In the saffron apical buds, the starch content was high in DS, slowly decreased by 11.33% from DS to BS and sharply decreased by 36.41% between BS and FS (Fig. 2A). In contrast, the soluble sugar content continuously increased by 65.14% from DS to FS (Fig. 2B).

**Figure 3:**
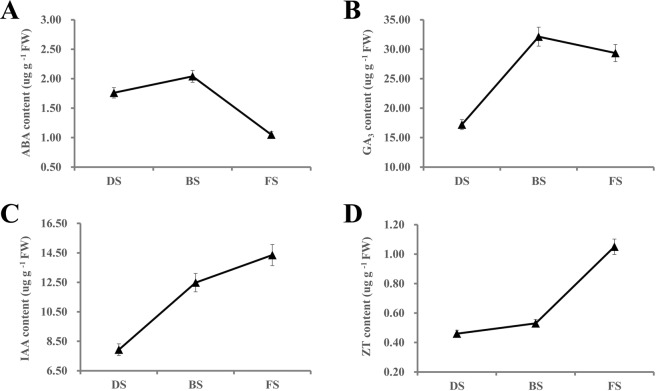
The hormone contents of apical buds during the flowering transition process in saffron. (**A**) Abscisic acid (ABA); (**B**) Gibberellin acid 3 (GA_3_); (**C**) Auxin (IAA); (**D**) zeatin (ZT).

The hormone contents were also analyzed in the apical buds at three stages during the flowering transition process (Fig. 3). The ABA content increased by 15.91% between DS and BS but sharply decreased by 48.53% from BS to FS (Fig. 3A). The GA_3_ content increased by 86.69% from DS to BS and slowly decreased by 8.65% between BS and FS (Fig. 3B). The IAA content continuously increased by 80.96% from DS to FS (Fig. 3C). In addition, the ZT content showed low levels between DS and BS but then sharply increased by 98.11% from BS to FS (Fig. 3D).

### General analysis of saffron transcriptome data

**Figure 4:**
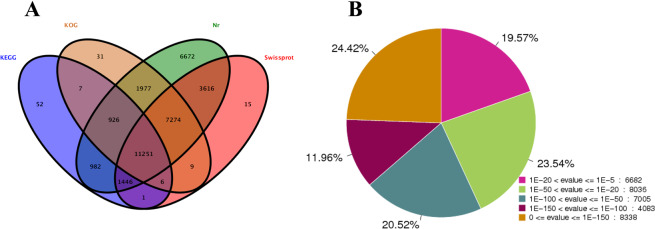
Annotation information of saffron unigenes. (**A**) Veen diagram of number of unigenes annotated by BLAXTx against protein databases; (**B**) E-value distribution of the top BLASTx hits against the nr database.

**Figure 5:**
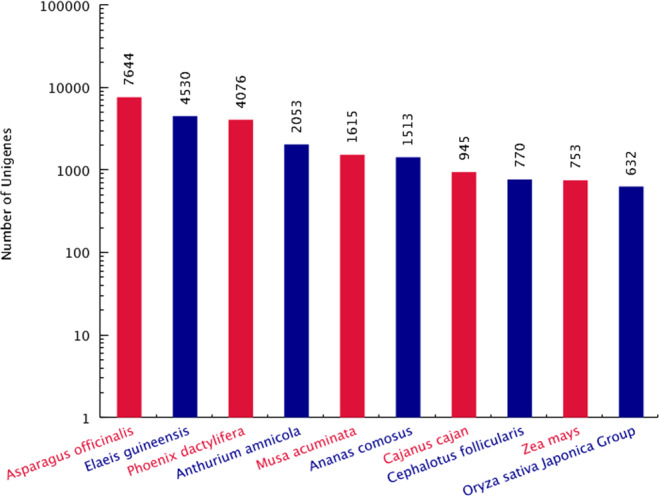
Number of unigenes matching the 10 top species using BLASTx in the nr database.

**Figure 6:**
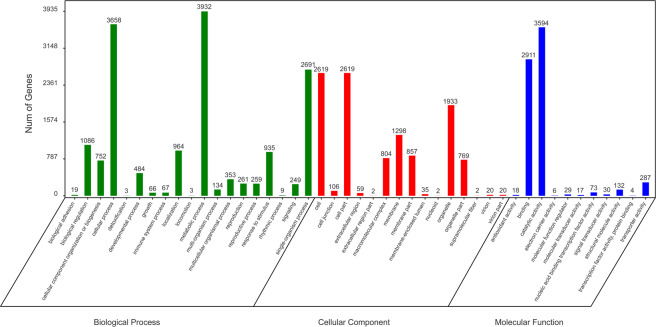
The GO classification of saffron unigenes.

In this study, a total of 430 853 316 raw reads were obtained from the three stages of saffron buds based on three biological duplications for each stage. After the elimination of low-quality reads and adaptor sequences, 422 584 176 clean reads were selected for further analysis (Table 1). Finally, 60 203 unigenes with a mean size of 1045 bp were assembled with lengths ranging from 201 to 14 704 bp (Fig. S1 of Appendix S1). The GC content and N50 of the unigenes were 43.49% and 1532 bp, respectively, indicating high assembly quality. Among the 60 203 unigenes, 34 144 (56.71%), 23 618 (39.23%), 21 481 (35.68%), and 14 671 (24.37%) unigenes were successfully annotated according to the NR, Swiss-Prot, KOG and KEGG databases, respectively (Fig. 4A). Based on the NR database, 19.57% of the unigenes showed homology (1e^−20^ < E-value <1e^–5^), 44.06% of those showed strong homology (1e^−100^ < E-value <1e^−20^) and the remaining 36.37% showed very strong homology (E-value <1e − ^100^) to the available plant sequences (Fig. 4B). As shown in Fig. 5, 16 250 unigenes were annotated to 3 top-hit species, *Asparagus officinalis*, *Elaeis guineensis* and *Phoenix dactylifera*. A total of 34 171 (56.76%) unigenes were successfully annotated using the gene ontology (GO) database and classified into 3 categories: biological processes (15 925), cellular components (11 145) and molecular functions (7101) (Fig. 6). In addition, 8251 unigenes were mapped into 130 standard pathways using the KEGG database (Table S1). The most abundant pathways were metabolic pathways (ko01100), with 2785 unigenes counted, followed by the biosynthesis of secondary metabolites (1332, 16.14%, ko01110), the biosynthesis of antibiotics (681, 8.25%, ko01130), ribosome (550, 6.67%, ko03010), starch and sucrose metabolism (323, 3.91%, ko00500), the plant hormone signal transduction pathway (294, 3.56%, ko04075), and ending with the limonene and pinene degradation pathway (1, 0.01%, ko00903).

**Table 1:** Throughput and quality of RNA-seq of DGE libraries.

Libraries	Raw Reads	Clean Reads (%)	Q20 (%)	Q30 (%)	GC (%)
DS-1	48676240	47860434 (98.32)	98.65	95.39	48.06
DS-2	44835112	43947582 (98.02)	97.97	93.69	48.26
DS-3	46206686	45191342 (97.80)	96.98	91.57	47.84
BS-1	38091200	37469968 (98.37)	98.76	95.70	48.05
BS-2	54333786	53224444 (97.96)	97.13	91.88	47.84
BS-3	49516516	48458008 (97.86)	97.12	91.87	47.99
FS-1	51857414	51052604 (98.45)	98.81	95.83	48.08
FS-2	54573954	53442128 (97.93)	97.14	91.90	47.94
FS-3	42762408	41937666 (98.07)	98.07	93.94	48.12

### DEG analysis of saffron transcriptome data

#### Identification of DEGs

**Figure 7:**
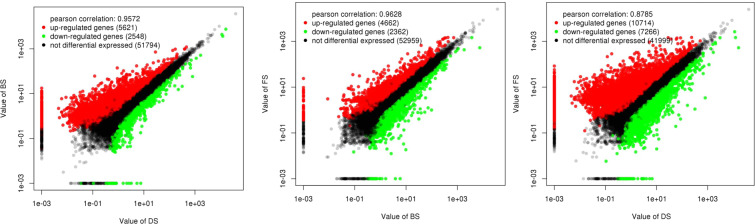
Volcano plot of the differentially expressed genes in DS vs BS, BS vs FS and DS vs FS.

**Figure 8:**
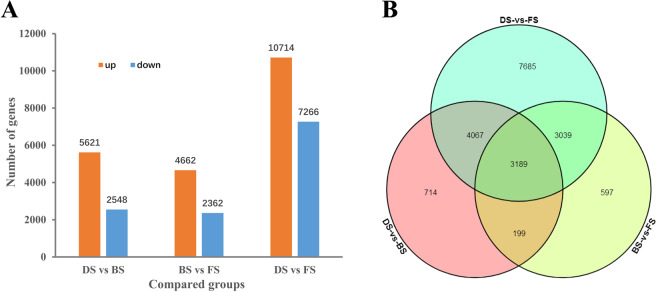
Numbers of DEGs in DS vs BS, BS vs FS and DS vs FS. (**A**) Column plot of the DEGs in each comparison; (**B**) Veen diagram analyses of DEGs in each comparison.

The repeatability of the 9 differential gene expression (DGE) libraries was evaluated using PCA and clustering analysis. The results showed that the three biological replicates at the DS, BS and FS stages could form a cluster, suggesting good repeatability between the replicates at the three stages (Fig. S2 of Appendix S1). Based on these analyses, differentially expressed genes (DEGs) were identified with an FDR (false discovery rate) <0.05 and an absolute value of log2-fold change ≥ 1. As a result, 5621 upregulated and 2548 downregulated unigenes between DS and BS were identified. Similarly, 4662 upregulated and 2362 downregulated unigenes and 10 714 upregulated and 7266 downregulated unigenes were obtained from BS to FS and DS to FS, respectively. (Fig. 7, and 8A). Most DEGs were identified for DS versus FS; 714, 597 and 7685 DEGs were found to be specifically expressed between DS and BS, BS and FS, and DS and FS, respectively (Fig. 8B).

#### GO functional analysis of DEGs

All DEGs between DS and BS, BS and FS and DS and FS were subjected to GO-term enrichment analysis. In total, 12 005 DEGs were classified into 3 main categories: biological processes, cellular components and molecular functions (Table S2). Under biological processes, a large number of DEGs were enriched for metabolic processes, cellular processes, and single-organism processes. In cellular components, the DEGs were mainly associated with the cell, cell part and organelle. For molecular functions, binding and catalytic activity were the most abundant subcategories (Fig. S3 of Appendix S1).

#### KEGG pathway analysis of DEGs

To characterize the expression profile of all the DEGs, the expression data υ (from DS to BS and BS to FS) were normalized to 0, log2 (BS/DS), and log2 (FS/DS). A total of 19 490 DEGs were clustered into 8 profiles based on an analysis using Short Time-series Expression Miner (STEM)^28^ (Fig. S4 of Appendix S1). A total of 14 402 genes, belonging to profiles 0, 6, and 7, showed highly significant differences (p-value < 0.001), and the remaining 5088 genes, belonging to profiles 1, 2, 3, 4, and 5, showed no significant differences (p-value > 0.05) from DS to BS and from BS to FS. Therefore, profiles 0, 6, and 7 were chosen for use in further analyses. Profile 0 was downregulated and contained 4514 DEGs, whereas profiles 6 and 7 were upregulated and contained 2774 and 7114 DEGs, respectively.

The DEGs were subjected to a KEGG pathway enrichment analysis. A total of 2531 unigenes were assigned to 128 standard pathways. The 10 top pathways with the highest representation of DEGs are shown in Table S3. Partial KEGG pathways associated with plant flowering transitions are listed in Table 2. The 5, 56 and 97 unigenes among the 393 (1.27%), 391 (14.32%) and 1319 (7.35%) DEGs, respectively, in profiles 0, 6 and 7 were annotated to the starch and sucrose metabolism pathways. The 13, 45, and 49 unigenes accounting for 3.31%, 11.51%, and 3.71% of genes, respectively, in profiles 0, 6, and 7 belonged to the plant hormone signal transduction pathway. One out of 393 unigenes in profile 0 (0.25%) and 6 out of 391 unigenes (1.53%) in profile 6 belonged to the Zeatin biosynthesis pathway, while no unigene in this pathway was detected in profile 7. In addition, 5 out of 393 unigenes in profile 0 (1.27%), 3 out of 391 unigenes (0.77%) in profile 6 and 6 out of 1319 unigenes (0.45%) in profile 7 were annotated to the circadian rhythm–plant pathway (the photoperiod pathway).

**Table 2:** Partial KEGG pathways associated with saffron flowering transition.

Pathway	No. of DEGs with pathway annotation				Pathway ID
	All profiles (2531)	Profile 0 (393)	Profile 6 (391)	Profile 7 (1319)	
Starch and sucrose metabolism	183 (7.23%)	5 (1.27%)	56 (14.32%)	97 (7.35%)	ko00500
Carbon metabolism	155 (6.12%)	16 (4.07%)	22 (5.63%)	102 (7.73%)	ko01200
Plant hormone signal transduction	127 (5.02%)	13 (3.31%)	45 (11.51%)	49 (3.71%)	ko04075
Spliceosome	67 (2.65%)	17 (4.33%)	5 (1.28%)	30 (2.27%)	ko03040
Carbon fixation in photosynthetic organisms	48(1.90%)	3 (0.76%)	9 (2.30%)	35 (2.65%)	ko00710
Fructose and mannose metabolism	30 (1.19%)	3 (0.76%)	6 (1.53%)	18 (1.36%)	ko00051
Pentose phosphate pathway	24 (0.95%)	3 (0.76%)	3 (0.77%)	18 (1.36%)	ko00030
Circadian rhythm - plant	21 (0.83%)	5 (1.27%)	3 (0.77%)	6 (0.45%)	ko04712
Photosynthesis	20 (0.79%)	6 (1.53%)	3 (0.77%)	5 (0.38%)	ko00195
Zeatin biosynthesis	10 (0.40%)	1 (0.25%)	6 (1.53%)	0 (0.00%)	ko00908

#### DEGs associated with saffron flowering transition

Table 3 shows the number of DEGs that were likely associated with the saffron flowering transition. The number of DEGs in the 3 significantly different expression patterns was calculated. A total of 165 unigenes were mainly involved in the sugar metabolism, plant hormone signal transduction, cell cycle regulatory, photoperiod (circadian rhythm-plant) and autonomous pathways (Table 3 and Appendix S2).

**Table 3:** Number of DEGs associated with saffron flowering transition.

Components	All profiles	Profile 0	Profile 6	Profile 7
**Sugar metabolism**				
*AMY*	3	0	1	2
*BAM*	4	0	1	3
*UGT*	4	1	1	2
*SUS*	10	1	4	5
**Plant hormone signal transduction**				
Auxin				
*AUX1*	7	0	2	5
*ARF*	7	1	3	3
*SAUR*	9	0	6	3
*AUX/IAA*	12	1	6	5
**Cytokinine**				
*CRE1*	4	1	2	1
*A-ARR*	5	2	2	1
*B-ARR*	14	0	9	5
**Gibberellin**				
*GID1*	2	0	0	2
*GAMYB*	1	0	0	1
*TF*	3	0	1	2
**Abscisic acid**				
*NCED*	5	5	0	0
*PYR/PYL*	3	0	0	3
*PP2C*	3	2	0	1
*ABF*	2	1	1	0
*CYP707A*	2	0	1	1
**Cell cycle regulatory**				
*CYCA/B/D*	21	0	8	13
*CDK*B	5	0	3	2
*KNOTTED*	4	0	2	2
**Circadian clock pathway**				
*PIF3*	3	0	1	2
*CHS*	3	0	1	2
*FT*	1	0	1	0
**Flowering activators**				
*FD*	4	1	1	2
*FLD*	1	0	0	1
*SOC1*	1	0	0	1
*AP1*	5	0	0	5
*AP2*	5	0	2	3
*AP3*	2	0	0	2
*PI*	4	0	2	2
*MADS-box*	6	2	1	3

In the photoperiod pathway, three unigenes were annotated as phytochrome interacting factors (*PIF3*), which play important roles in plant germination, morphogenesis and hormonal signal transduction^29^. One of them belonged to profile 6, and two belonged to profile 7. Additionally, three unigenes annotated as chalcone synthases (*CHS*) were also assigned to profiles 6 and 7. One unigene encoding *FT* was attributed to profile 6. In total, 7 DEGs belonging to the photoperiod pathway were significantly upregulated from DS to FS and induced the saffron floral transition (Table 3 and Appendix S2).

In the autonomous pathway, *FLD* could control the flowering time and activate flowering by restraining the expression of *FLC* for flowering inhibitors^30^ and was clustered in profile 7. It was expressed at a low level in the DS stage but was significantly expressed in the FS stage (Table 3). In addition, one unigene encoding *FRI* and three unigenes encoding *DRM1* were found to be differentially expressed between DS and FS and were also involved in the saffron flowering transition (Appendix S2).

In the sugar metabolism pathway, 10 unigenes encoding sucrose synthase (*SUS*) were found to be differentially expressed, among which four were clustered to profile 6 and five to profile 7. One unigene belonging to profile 6 and two unigenes belonging to profile 7 were annotated as UDP-glycosyl transferase (*UGT*). In addition, *AMY* and *BAM*, which are associated with starch metabolism, were also clustered in profile 6 or 7. These DEGs showed similar upregulation trends between DS and BS and positively regulated saffron floral transduction (Table 3 and Appendix S2).

In the cell cycle regulatory pathway, we identified three cyclin (*CYC*), cyclin-dependent kinase (*CDKB*) and *KNOTTED-like* genes. A total of 30 unigenes belonging to profile 6 or 7 were expressed at a low level in the DS stage but were highly increased in the FS stage. The DEGs involved in the cell cycle regulatory pathway could promote the saffron flowering transition (Table 3 and Appendix S2).

In the auxin signal transduction pathway, 7 unigenes annotated as auxin influx transport proteins (*AUX1*) were found to be differentially expressed, two of which were assigned to profile 6, and five to profile 7, showing different upregulated expression patterns. It was found that 7 unigenes encoded auxin response factor (*ARF*). Six of them were clustered in profile 6 or 7, showing upregulation trends, and one was clustered to profile 0, showing a downregulation trend. Furthermore, nine unigenes encoding indole-3-acetic acid-induced protein (*SAUR*) were also assigned to profile 6 or 7. The expression level was relatively high in the FS stage. Eleven out of the 12 unigenes encoding auxin-induced protein (*AUX/IAA*) showed upregulation trends, and 1 showed a downregulation trend. In other words, genes with upregulation trends were more abundant than those with downregulation trends, which was similar to the results of the cytokinin and gibberellin transduction pathways. This result suggests that the DEGs involved in the auxin signal transduction pathway positively regulated the saffron floral transition (Table 3 Appendix S2).

In the cytokinin signal transduction pathway, 1 unigene encoding a cytokinin receptor (*CRE1*) and 2 encoding type-a response regulators (*A-ARR*) showed patterns of downregulation, while 14 unigenes encoding type-b response regulators (*B-ARR*) showed patterns of upregulation. In the gibberellin signal transduction pathway, 2 and 1 unigenes were identified with upregulation profiles and annotated as gibberellin receptor (*GID1*) and *GAMYB*, respectively. In contrast, 2 unigenes encoding gibberellin 2-beta-dioxygenase (*GA2ox*) were identified with downregulation profiles, which could catalyze the 2-beta-hydroxylation of gibberellin precursors, rendering them unable to be converted to active GAs (Table 3 and Appendix S2).

In the abscisic acid signal transduction pathway, 8 out of the 15 DEGs were clustered to profile 0, showing downregulation trends. They encoded 9-cis-epoxycarotenoid dioxygenase (*NCED*), type-2C protein phosphatase (*PP2C*) and ABA responsive element binding factor (*ABF*), among which *NCED* could positively regulate ABA biosynthesis^31^. Three unigenes encoding abscisic acid receptors (*PYR/PYL*) and 2 unigenes encoding abscisic acid 8’-hydroxylase (*CYP707A*) were assigned to profile 6 and profile 7, respectively and were upregulated between DS and FS. *CYP707A* is a key enzyme for ABA dissimilation^32^. These results indicated that abscisic acid may play a negative role in the saffron floral transition (Table 3 and Appendix S2).

### Verification of DEG expression by qRT-PCR

**Figure 9:**
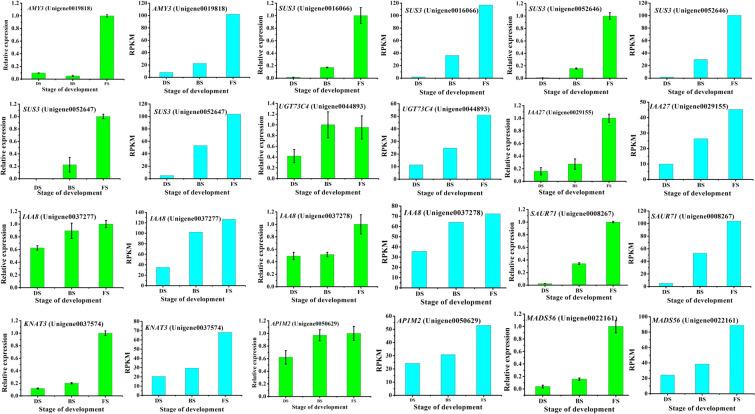
Verification of 12 flowering transition-related DEGs by qRT-PCR.

To verify the accuracy and reproducibility of the transcriptome analysis results, 12 unigenes were selected for qRT-PCR validation (Fig. 9), including *AMY3* (Unigene0019818), *SUS3* (Unigene0016066), *SUS3* (Unigene0052646), *SUS3* (Unigene0052647), *UGT73C4* (Unigene0044893), *IAA27* (Unigene0029155), *IAA8* (Unigene0037277), *IAA8* (Unigene0037278), *SAUR71* (Unigene0008267), *KNAT3* (Unigene0037574), *AP1M2* (Unigene0050629) and *MADS56* (Unigene0022161). The results showed that the expression patterns of the candidate unigenes revealed by qRT-PCR were in good agreement with those derived from RNA-Seq, indicating the reliability of the RNA-Seq data.

## Discussion

### Sugar signaling mediates the flowering transition in the saffron

Starch is the most important form of nutrient reserve in plants. Its content decreased gradually from BS to FS (Fig. 2A), while the soluble sugar content showed a gradual upregulation trend (Fig. 2B), indicating that abundant soluble sugar was necessary for the flowering transition in saffron. Similar results have been obtained in other plants^33,34^. The partial DEGs involved in the sugar metabolism of saffron are shown in Appendix S3. Most of them were significantly upregulated, such as *AMY*, *BAM*, UGT, *SUS*, and *MSSP2*, which is consistent with the sugar content changes during the flowering transition process. Trehalose-6-phosphate (T6P) is considered a signal substance for sufficient allocation of carbohydrates in plants and plays a key role in inducing flowering^35^. *SPLs* are viewed as floral activators, which could be inhibited by miRNA156. However, T6P represses the expression of miRNA156 and indirectly activates *SPLs* to promote flowering^36^. In addition, T6P can directly influence the expression levels of *FT* genes to accelerate the flowering transition process^37^. Our results showed that *TPS* genes involved in T6P biosynthesis and *TPP* genes involved in the T6P degradation process were upregulated and downregulated, respectively (Appendix S3). Meanwhile, some flower integrators, i.e., *SPL*, *FT*, *FD* and *AP1*, were highly expressed during the floral transition process (Table 3 and Appendix S2). This suggests that sugar signaling may participate in flower induction via the T6P pathway.

### Hormone signaling mediates the flowering transition in saffron

Gibberellin (GA) is considered to be the most important category of hormones in plant flowering regulation, and they play a positive role in flowering in *Arabidopsis*^38^. Previous studies have demonstrated that GAs promote flowering by increasing the expression of genes such as *LFY*, *TSF*, *SOC1*, *FT*, and *SPL*, while this effect is inhibited by the DELLA protein. In this study, *GA2ox*, which catalyzes bioactive GAs into inactive forms, was found to be downregulated from DS to FS. *SPY*, which activates the DELLA protein by O-GlcNAc modification, showed a similar downregulation trend. In contrast, the *GID1*, *GID2* and *TF* genes, which are involved in the gibberellin signal transduction pathway, and the *SOC1*, *AP1*, and *SPL* genes, which are related to floral induction, were upregulated from DS to FS, suggesting that gibberellin plays a vital role in the flowering transition of saffron. *GAMYB*, a downstream component of the gibberellin reaction, binds to the promoter of the floral meristem-specific gene *LFY* and then enhances *LFY* expression. Furthermore, *GAMYB* could also improve the synthesis and activity levels of α-amylase. Indeed, our data showed that the expression levels of the *GAMYB* and *AMY* genes were significantly upregulated from DS to FS (Table 3 and Appendix S2), indicating that GAs may regulate floral transition in saffron by promoting flowering-related gene expression or interacting with starch metabolism pathways. In the studies of flowering induction in Chrysanthemums^39^ and *Angelica sinensis*^40^, we also found that the Gibberellin pathway was involved in the flowering. However, the results of our physiological indicators showed that the GA_3_ content did not increase significantly in the FS stage (Fig. 3B). According to other reports, GA_4_ is the most active type of GA in *Arabidopsis* flowering induction^41^. Thus, we speculate that other types of GAs might mediate the saffron flowering transition, such as GA_1_, GA_2_, GA_4_, and GA_7_. It will be interesting to study the levels of other types of GA in saffron to verify these results.

Cytokinin (CTK), a vital phytohormone involved in regulating the dynamic balance between the cell division cycle and meristems, participates in many aspects of plant growth and development, including the growth of shoot apical meristems^42^, the regulation of the transition from vegetative growth to reproductive growth and the flowering induction process^43^. Indeed, our results showed that the level of ZT and *CRE1*, *A-ARR*, and *B-ARR* genes, which are related to the cytokinin signal pathway, were synchronously upregulated from BS to FS (Fig. 3D and Table 3), and *CKX11*, as a CK degradation gene, showed a downregulation trend (Appendix S2), indicating that cytokinin-related genes are involved in the flowering transition in saffron. Similar observations have been reported in apple (*Malus domestica* Borkh.)^35^. Moreover, the high expression levels of *CYCA*, *CYCB*, *CYCD*, *CDKB* and *KOTTED* and the low expression level of *CKI* in the FS stage (Table 3 and Appendix S2) suggest that cytokinin positively mediates the process of flower induction regulation in saffron. Auxin, which plays a pivotal role in regulating the flowering transition, has also been widely reported^44,45^. In our data, the level of auxin and DEGs, *AUX1*, *ARF*, *SAUR* and *AUX/IAA* had significant differences between DS and FS (Table 3 and Appendix S2), indicating that auxin-related genes participate in the flowering transition of saffron. However, further experiments are necessary to confirm the specific regulatory mechanism.

In this study, ABA levels gradually decreased in the buds during the flowering induction process (Fig. 3A), and several genes related to ABA biosynthesis and signal transduction, including *NCED*, *PP2C* and *ABF*, displayed similar changes. The ABA degradation gene *CYP707A* showed an opposite trend from DS to FS (Table 3 and Appendix S2), suggesting that ABA may play a negative role in the saffron floral transition. This is contrary to the result that ABA could positively mediate the floral induction of *Litchi chinensis*^46^, which may be due to different regulatory mechanisms of ABA in different plant flower induction processes. *SnRK1* not only is a crucial component of the ABA signaling pathway but also participates in sugar metabolism^47^, indicating that ABA may interact with sugars to mediate the flowering transition. Additionally, ABA could affect the expression of *FCA*, an ABA binding protein, and restrain flowering^48^.

### The flowering pathway in saffron during the floral transition

It has been widely reported that the flowering transition is actually a complex physiological and morphological change in response to internal (sugar, GAs, age and autonomous) and environmental (photoperiod and vernalization) signals in *Arabidopsis*^37^. *PIF3* is a signal factor that interacts with photoactivated phytochrome and transmits light signals to the downstream circadian clock-controlled gene *CO* and activates its expression^29^. In our data, the *PIF3*, *COL*, *CHS*, and *FT* genes, which are related to the photoperiod (circadian rhythm), increased from BS to FS, while *CDF1*, which inhibits the expression of *CO*, was downregulated (Appendix S2), indicating that the photoperiod positively mediates the saffron flowering transition. Similar observations have been reported in *A. sinensis*^40^ and *Juglans regia*^49^.

The autonomous pathway is another vital regulation mechanism, which includes *FLD*, *FCA*, *LD*, *FY*, *DRM1*, and *FRI* and generally repress the expression of the *FLC* gene to promote flowering^50^. *FLC*, a key flowering inhibitor, binds to the first intron of *FT* and the promoters of *FD* and *SOC1* genes, and represses the expression of those genes^51^. During the saffron flowering transition, the *FRI* gene is significantly downregulated at the FS stage (Appendix S2), which could activate *FLC* gene expression. Other autonomous pathway genes, such as *FLD*, *DRM1*, *FT* and *SOC1*, were all upregulated from DS to FS (Appendix S2). However, *FLC* was not detected among the expressed genes of the flowering transition in saffron, indicating that the autonomous pathway may regulate saffron flowering in other ways. Further experiments are necessary to refine the regulatory mechanism. Interestingly, in *Angelica sinensis*, also used as a medicinal plant, we found that all key genes in the autonomous pathway are not changed when it flowers^40^. There are similarities in the gene expression associated with flowering in AS and saffron, but there are still some differences in gene expression at the same time. These different genes will be the focus of our future research work.

Vernalization is the acceleration of flowering by exposing a plant to cold conditions for a long time. Saffron is an autumn-flowering plant that does not need a cold environment to blossom, so we speculated that saffron flower induction is not influenced by vernalization. In fact, we did not obtain any genes related to the vernalization pathway in the transcriptomic data, such as *VIN1*, *VIN2*, *VIN3*, *VRN1* and *VRN2*. In contrast, the vernalization pathway is important for floral transition in *A. sinensis*^40^, *blueberry*^52^ and *Paeonia suffruticosa*^53^.

The above pathways involved in the flowering induction of saffron eventually converge towards the floral meristem, where the *SOC1*, *LFY*, *AP1*, *AP2* and *AP3* genes irreversibly contribute to the transition from vegetative growth to reproductive growth. Once *SOC1* is activated, the expression of *LFY*, a meristem determine gene, is promoted. Subsequently, *LFY* induces *AP1* gene expression to initiate the flowering transition. In this study, the floral organ-determining genes, including *AP1*, *AP2*, *AP3* and *PI*, were significantly upregulated from DS to FS (Table 3 and Appendix S2), suggesting that the saffron had begun to enter the flower bud differentiation stage. Additionally, this study also found that the expression levels of several *MADS-box* family genes were synchronously upregulated during the saffron flowering transition (Appendix S2), indicating that the *MADS-box* genes have a positive effect on saffron floral induction.

**Figure 10:**
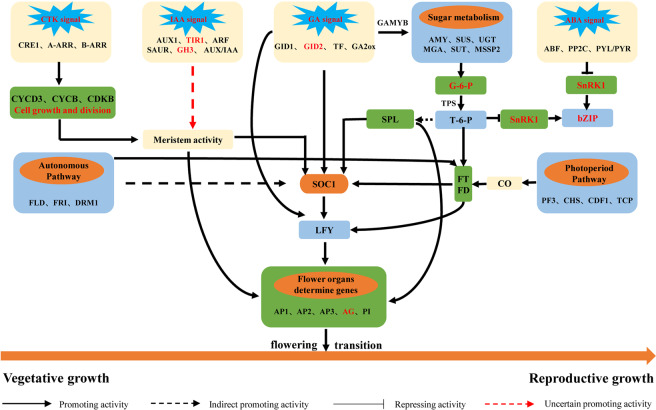
Hypothetical model for the molecular regulatory networks of flowering transition in the saffron (Genes in black font indicate “real genes” which were supported by the sequence data in this study, while genes in red font indicate “novel genes” which were supported by other researches, but not detected among the expressed genes of the flowering transition in saffron).

Overall, the flowering transition of *C. sativus* is a process of complex morphological and physiological changes and might be regulated by the sugar metabolism, hormone signal transduction, cell cycle, circadian rhythm and autonomous pathways. We proposed a regulatory network for the saffron flowering transition encompassing an overview of the known floral regulators present and differentially expressed genes during the flowering transition of saffron (Fig. 10), which provides a foundation for further research on the flowering regulatory mechanism of saffron.

## Materials and methods

### Plant material

Saffron corms (15–20 g) were planted in the medicinal botanical garden of Chengdu University of Traditional Chinese Medicine and managed conventionally. Apical bud samples along with a portion of surrounding tissue from the corms (Fig. 1: red circle) were collected on May 29 (DS stage), July 10 (BS stage) and August 15 (FS stage) in 2018. Some of the samples were immediately frozen in liquid nitrogen and stored at −80 °C for RNA-seq and RT-qPCR, and the levels of starches, sugars, and hormones were measured. The remaining samples were stored in FAA fixative (5:6:89 ratio of formalin: glacial acetic acid:50% ethanol) for morphological observation.

### Morphological observations

Apical bud tissues at the three developmental stages were collected and fixed quickly in FAA solution. The fixed samples were dehydrated with a graded ethanol series (50–100%), embedded in paraffin and sliced to a 10-µm thickness. The dried slices were deparaffinized with xylene, hydrated in a gradually decreasing ethanol series, and stained with Safranin and Fast Green for 30 s. Finally, the slices were sealed with neutral gum and then observed and photographed using a Leica DM2000 optical microscope.

### Measurements of starch, sugar and hormone contents

The total soluble starch and sugar contents at the three developmental stages, namely, DS, BS, and FS, were measured by the sulfuric acid-anthrone colorimetric method^54^. Three biological replicates for each stage and three technical replicates for each biological replicate were obtained.

Hormone extraction was primarily conducted according to the methods from a previous report with some modifications^55^, and the specific hormone extraction process is shown in Fig. S5 of Appendix S1. Three biological replicates for each stage and three technical replicates for each biological replicate were obtained. The resulting solutions, F1 and F2, were filtered through 0.45 μm regenerated cellulose membrane syringe filters and directly injected into an HPLC system (Agilent 1200, USA). Separations were carried out in a C18 column (5 µm, 4.6 mm × 250 mm, Agilent Zorbax Eclipse XDB-C18, USA). The mobile phase consisted of solvent A (water with 0.6% glacial acetic acid) and solvent B (acetonitrile). A gradient elution was utilized for the extraction solution (F1) as follows: 0–15 min, 15% B; 15–40 min, 15% - 58% B; 40–45 min, 100% B; and 45–60 min, 15% B. The extraction solution (F2) was isocratically eluted with solvent A and solvent B (86:14). The mobile phase flow rate was 1.0 mL/min, the UV absorption was monitored at 270 nm, the column temperature was maintained at 35 °C, and the sample injection volume was 20 μL.

### RNA extraction, library construction and sequencing

The RNA of the apical bud tissues from the three developmental stages, DS, BS, and FS (three replicates for each stage), was extracted using an RNAprep Pure Plant Kit (Polysaccharide- & Polyphenolic-rich) (TIANGEN, Beijing, China) according to the manufacturer’s protocol, and DNA contamination was removed with RNase-free DNase I (Takara, Dalian, China). The purity, concentration and integrity (RIN) of the RNA were determined using a NanoPhotometer spectrophotometer (IMPLEN, CA, USA), a Qubit@ RNA Assay Kit with a Qubit R 2.0 Fluorometer (Life Technologies, CA, USA) and an RNA Nano 6000 Assay Kit with an Agilent Bioanalyzer 2100 system (Agilent Technologies, CA, USA), respectively.

To identify DEGs during the saffron flowering transition, the RNA from the 9 samples (three replicates for each stage) was used to construct libraries for digital gene expression (DGE) profiling analyses. The libraries were as follows: DS-1, DS-2 and DS-3 as replicate libraries for the undifferentiated period; BS-1, BS-2 and BS-3 for the early flower bud differentiation period; and FS-1, FS-2 and FS-3 for the late flower bud differentiation period. A transcriptome assembly reference library was constructed by mixing equal amounts of RNA from the above 9 samples. In short, total mRNA was isolated with Oligo (dT) cellulose and then fragmented and reverse transcribed with random primers. Second-strand cDNA was synthesized by DNA polymerase I, RNase H, dNTP and a buffer. Then, the cDNA fragments were purified with a QiaQuick PCR extraction kit, end repaired, poly(A) added, and ligated to Illumina sequencing adapters. The ligation products were size selected by agarose gel electrophoresis, fragments were excised for PCR amplification, and the amplified fragments were sequenced using an Illumina HiSeq. 4000 from Gene Denovo Biotechnology Co. (Guangzhou, China).

### *De novo* assembly and functional annotation

After sequence data were obtained, raw reads were filtered by removing adapter sequences, reads containing more than 10% of unknown nucleotides, and low-quality reads with more than 40% of low Q-value (≤10) bases. The raw data were uploaded to the NCBI (Submission ID: SUB5330397). Then, de novo assembly based on the clean reads data was performed using the Trinity program^56^, and the final sequences of the Trinity assembly were defined as unigenes. All unigene sequences were aligned by Blastx with an E-value threshold of 1e^−5^ to protein databases, including the NCBI nr database (http://www.ncbi.nlm.nih.gov), the Swiss-Prot protein database (http://www.expasy.ch/sprot), the KEGG database (http://www.genome.jp/kegg), and the KOG database (http://www.ncbi.nlm.nih.gov/COG/KOG). Protein functional annotations of the unigenes could then be acquired according to the best alignment results. GO annotation of the unigenes was analyzed by Blast2GO software based on the nr annotation information^57^, and functional classification of the unigenes was performed by using WEGO software^58^.

### Differential gene expression analysis

The RPKM (Reads Per kb per Million reads) method was used to calculate the expression of the unigenes^59^. Then, differential expression analysis between two groups was performed by using edgeR (Bioconductor version 3.2.4). The resulting p-values were adjusted according to Benjamini and Hochberg’s approach for controlling the false discovery rate (FDR)^60^. In this experiment, an adjusted p-value (q-value) <0.05 and |log2-fold change| ≥ 1 were adopted to identify the DEGs between each comparison. In addition, gene expression data υ (from the DS to the FS stage) were normalized to 0, log2 (BS/DS), and log2 (FS/DS), and DEGs were clustered by using STEM software^28^. The clustered profiles with p-values ≤ 0.05 were considered to be significantly expressed. Then, the DEGs were subjected to GO functional classification and KEGG pathway enrichment analyses^61,62^.

### Quantitative Real-Time PCR Confirmation of the RNA-Seq Data

Twelve DEGs were randomly selected for quantitative real-time PCR (qRT-PCR) to validate the results of RNA-seq, and the saffron tubulin gene, *TUBA*, was selected as a reference gene. Specific primers for qRT-PCR were designed using Primer Premier 5.0 software (Premier, Canada) and synthesized by Tsingke Biotech (Chengdu) Co., Ltd. All the primer information is listed in Table S4. RT-qPCR was conducted using the Bio-Rad CFX96 Real Time PCR System (Bio-Rad, USA) and the SYBR Green-based PCR assay. Each PCR mixture had a volume of 20 μL containing 1 μL of cDNA as a template, 1 µL of each forward and reverse primer, 7 µL of ddH2O and 10 µL of SYBR Green PCR Master Mix (TaKaRa, Japan). The amplification program was as follows: 95 °C for 3 min, followed by 43 cycles at 95 °C for 10 s each, 58 °C for 30 s, and 95 °C for 10 s in 96-well optical reaction plates (Bio-Rad, USA). To determine the amplification specificity and the presence of reaction contaminants, a melting curve was generated for each PCR by using the Bio-Rad PCR System. The melting curve was obtained by heating the amplification products from 65 °C to 95 °C in 5 s intervals. Then, the primer efficiency was analyzed by CFX Manager Software v3.1 (Bio-Rad). Each qRT-PCR reaction was performed in three replicates, and the relative expression levels of the candidate genes were calculated by the 2^−ΔΔCt^ method^63^.

## Supplementary information

Supplementary Information.

Supplementary Information 2.

Supplementary Information 3.

Supplementary Information 4.

Supplementary Information 5.

Supplementary Information 6.

Supplementary Information 7.
