# Supplementary material for: Transcriptome profiling of the flowering transition in saffron (Crocus sativus L.)
Source: Sci Rep. 2020 Jun 15;10:9680. doi: 10.1038/s41598-020-66675-6 (PMC7295807; doi:10.1038/s41598-020-66675-6)

Transcriptomic analysis reveals multiple pathways coregulating the flowering transition in saffron (*Crocus sativus* L.)

Jing Hu[1], Yuping Liu[1], Xiaohui Tang[1], Huajing Rao[1], Chaoxiang Ren[1], Jiang Chen[1], Qinghua Wu[1], Yi Jiang[2], Fuchang Geng[3] and Jin Pei[1*]

[1]State Key Laboratory of Traditional Chinese Medicine Resources Research and Development, Chengdu University of Traditional Chinese Medicine, Chengdu 611137, China

[2]New Zealand Academy of Chinese Medicine Science, Christchurch 8014, New Zealand

[3]The Good Doctor Pharmaceutical group co. LTD, Mianyang 622650, China

*Corresponding author e-mail: peixjin@163.com

Fig. S1: The length distribution of saffron Unigenes

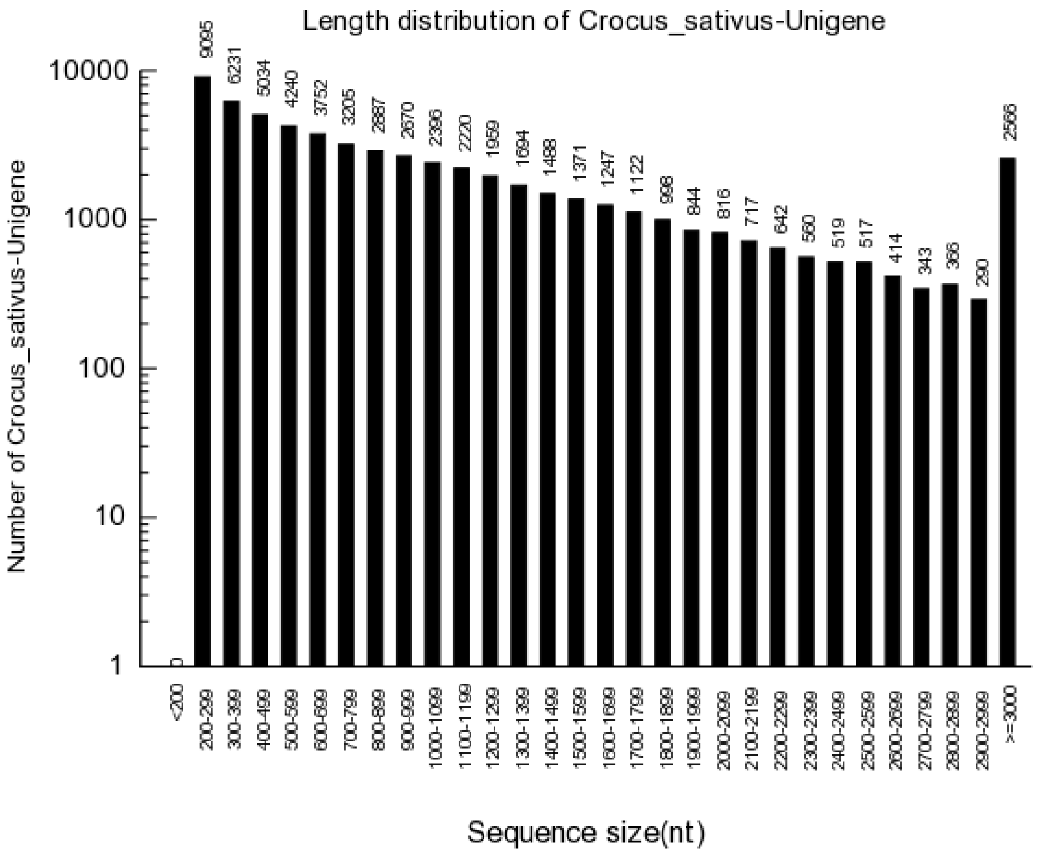

Fig. S2: The PCA and clustering analysis of three biological replicates at three stages

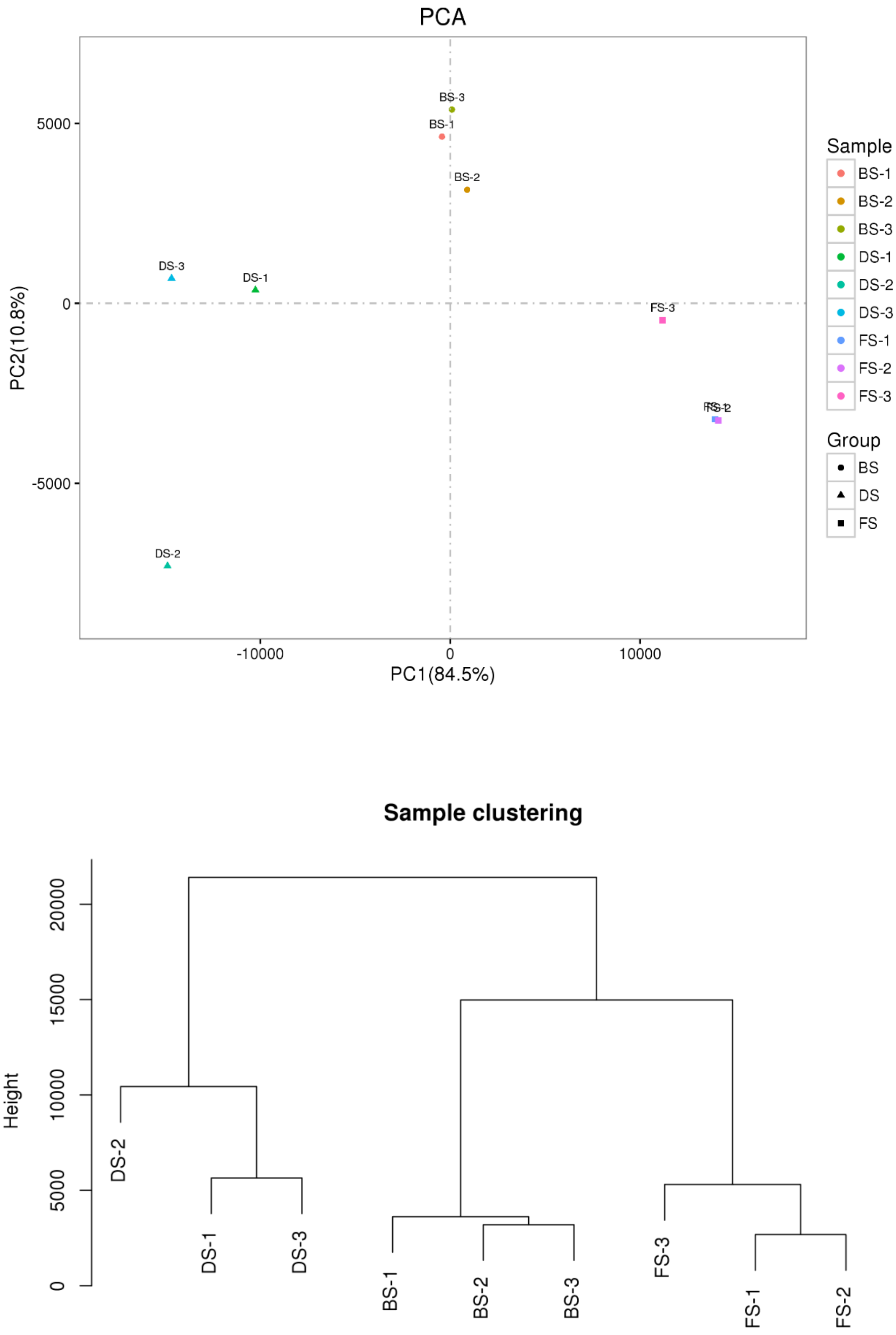

Fig. S3: The GO classification of all the DEGs

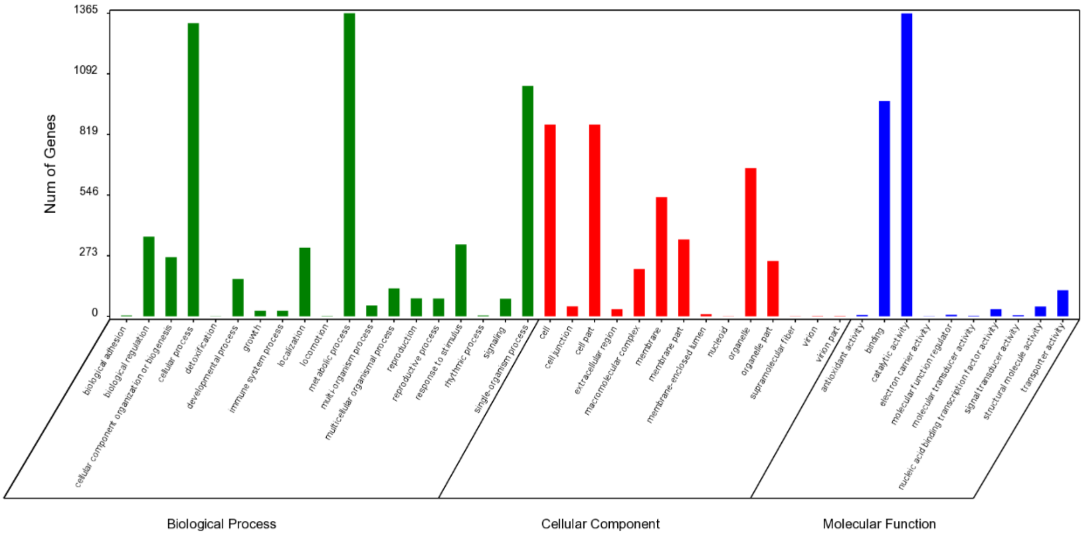

Fig. S4: The expression profile of all the DEGs

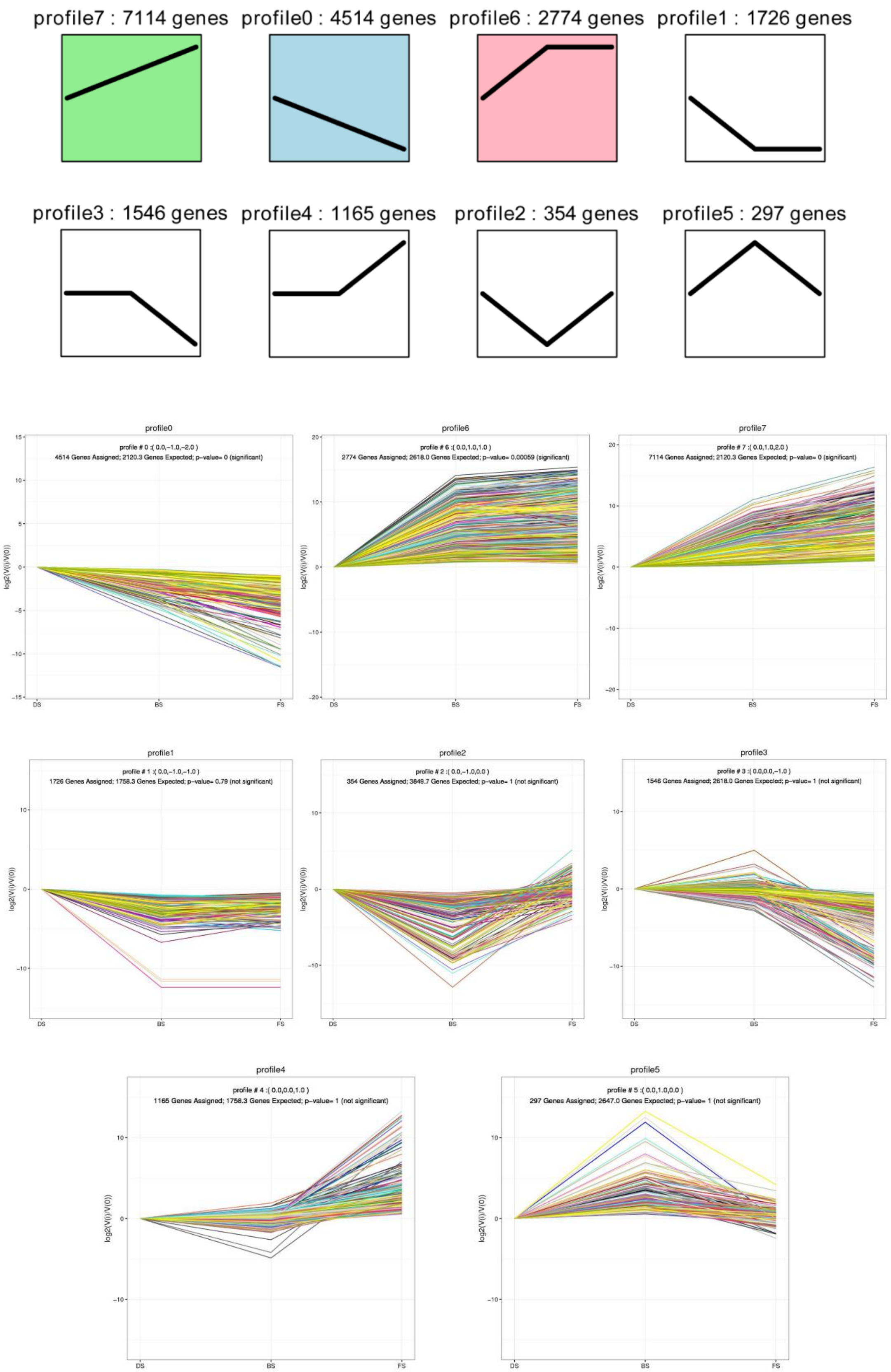

Fig. S5: The specific extraction process of hormone

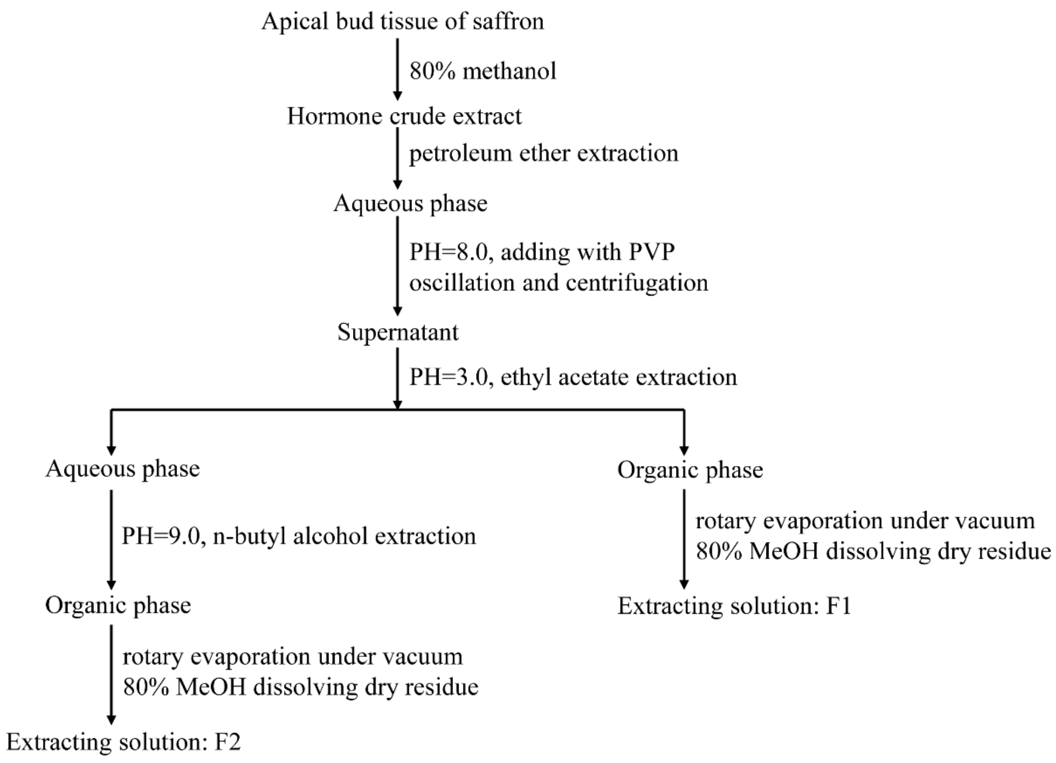

Supplement: Supplementary file 1 — Supplementary Information. [file 41598_2020_66675_MOESM1_ESM.pdf]
