# Supplementary figures and images for: Transcriptome profiling of the flowering transition in saffron (Crocus sativus L.)
Source: Sci Rep. 2020 Jun 15;10:9680. doi: 10.1038/s41598-020-66675-6 (PMC7295807; doi:10.1038/s41598-020-66675-6)

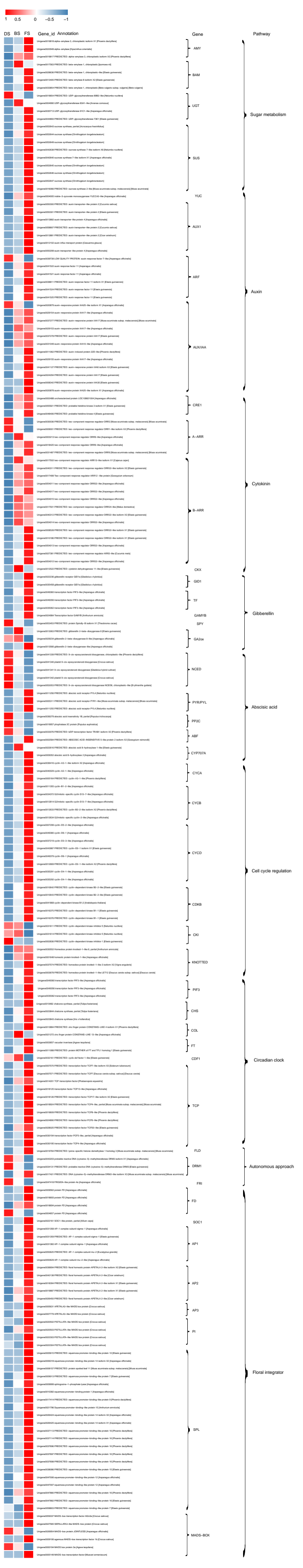

Supplement: Supplementary file 2 — Supplementary Information 2. [file 41598_2020_66675_MOESM2_ESM.pdf]

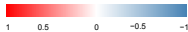

DS BS FS

Gene\_id Annotation

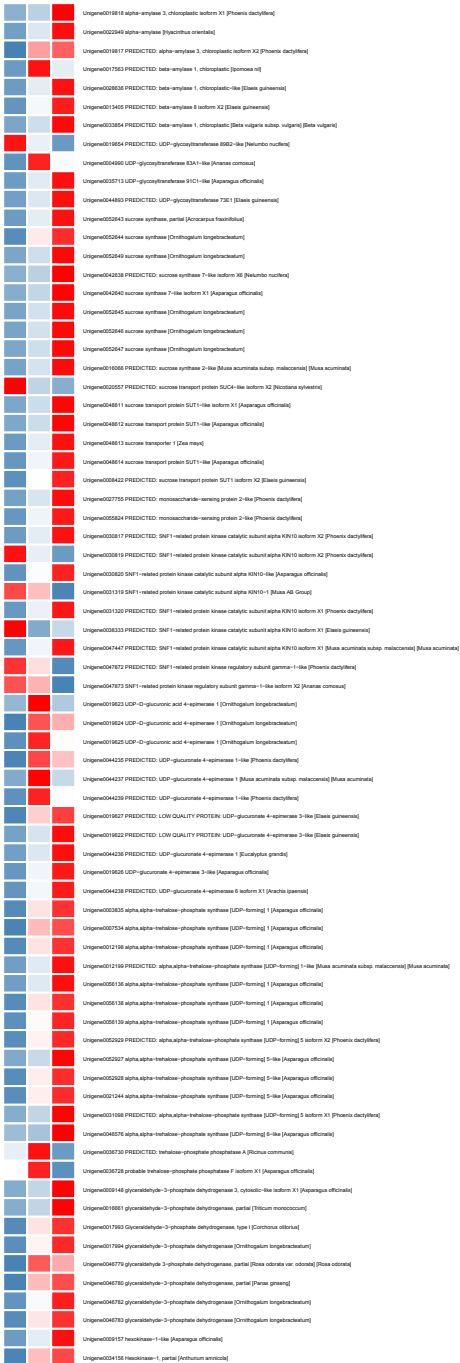

gene

AMY

BAM

UGT

SUS

SUT

MSSP2

KIN10

KING1

GAE

TPS

TPP

GAPC

HXK

Pathway

Supplement: Supplementary file 3 — Supplementary Information 3. [file 41598_2020_66675_MOESM3_ESM.pdf]
